# Morphological parameters of fourth lumbar spinous process palpation: a three-dimensional reconstruction of computed tomography

**DOI:** 10.1186/s13018-020-01750-2

**Published:** 2020-06-22

**Authors:** Qi Feng, Lei Zhang, Mengyao Zhang, Youliang Wen, Ping Zhang, Yi Wang, Yan Zeng, Junqiu Wang

**Affiliations:** 1 School of Clinical Medicine, Southwest Medical University, Luzhou, China; 2 Academician Workstation in Luzhou, Luzhou, China; 3 Department of Orthopedics, Affiliated Traditional Chinese Medicine Hospital of Southwest Medical University, Luzhou, 646000 China; 4 National Key Discipline of Human Anatomy, School of Basic Medical Sciences, Southern Medical University, Guangzhou, China; 5 School of Rehabilitation Medicine, Gannan Medical University, Ganzhou, China; 6 Operating Room, Affiliated Traditional Chinese Medicine Hospital of Southwest Medical University, Luzhou, China; 7 School of Chinese and Western Clinical Medicine, Southwest Medical University, Luzhou, China; 8 Department of Nephropathy, Affiliated Traditional Chinese Medicine Hospital of Southwest Medical University, Luzhou, China

**Keywords:** Palpation, Spine, Lumbar vertebrae, Imaging, Three-dimensional

## Abstract

**Background:**

The localization of lumbar fourth spinous process (L4-SP) is an important anatomical landmark, and identifying its accurate position is essential for the diagnosis and treatment of waist diseases.

**Methods:**

Five hundred participants were scanned with positive and lateral computed tomography (CT), which aimed to clarify anatomic characteristics of L4-SP. Anatomical parameters of the surface localization of L4-SP were measured and recorded through a three-dimensional (3D) reconstruction.

**Results:**

Five hundred participants were classified into three types according to the position of BC with the iliac spine. There are just 266 that the line between the highest point of the iliac spine on both sides located on L4-SP (type I, 53.20%), 16 above L4-SP (type II, 3.20%), and 218 below L4-SP (type III, 43.60%). BC in type I (15.92 ± 1.30 mm) is longer than type III (15.56 ± 1.32 mm). While the angle combined with AB and BC is different in the three groups, the angle in type I (173.00 ± 4.83°) is larger than that in type II (164.69 ± 5.50°) and type III (159.45 ± 8.39°). Other measurements were not found any significant differences between above.

**Conclusion:**

The traditional palpation for L4-SP is not absolutely exact. The accuracy rate is only 53.20%, and the errors may cause serious consequences.

## Background

Lumbar fourth spinous process (L4-SP) is in the connection between the highest point on both sides of the posterior superior iliac spine (PSIS) with its surface localization relates to the examination, diagnosis, and treatment of many lumbar diseases [1]. Clinical operations often find body position line—the highest point on both sides of PSIS to find the exact location of L4 [2]. Some scholars deal with L4-SP are performed frequently, including lumbar puncture and spinal anesthesia (SA). The results of degenerative changes of lumbar intervertebral disk increased with age until the incidence rate over 90% after the age of 50, and at the same time, lumbar disk herniation has a lifetime incidence of 1-2%, especially in the L4-L5 and L5-S1 [1, 3–5]. After that, both conservative treatment and surgical treatment of lumbar disk herniation are based on the exact location of L4.

Lumbar puncture is wildly used in the central nervous system; the entry point of lumbar puncture is the line determined by the superior part of the iliac crest, and inserting the needle at the L3/4 or L4/5 intervertebral space is usually as an accepted practice. In addition, due to the inter-individual deviation of L4-SP, the identification of suitable approaches to the entry point is challenging, especially newborns, the elderly, and parturients [6, 7].

Furthermore, spinal anesthesia is a form of regional anesthesia involving injection of a local anesthetic into the subarachnoid space, which is a commonly used anesthetic technique, both alone and in combination with either sedation or general anesthesia. It also depends on the accurate surface location of L4-SP. Local anesthetics were injected into the subarachnoid space through the L3/L4 intervertebral space to block the conduction function of some spinal nerves and induce anesthesia in the corresponding dominant areas. Anatomically, these operations go from shallow to deep through the skin, spinous ligaments, interspinous ligament, ligamentum flavum, epidural space, and dura mater spinalis, with nerves surrounded. Above that, the wrong positioning will hurt these structures can lead serious consequences including paraplegia and headache. In that case, it was clear that adequate knowledge of L4 morphology is necessary for the spinal surgeon in order to avoid damage to the vertebral arteries, spinal cord, or nerve roots during fixation interventions involving the posterior cervical spine. However, the number of failed lumbar puncture cases remains high [6, 8, 9]. Among the rest, postdural puncture headache is the most common complication, which is caused by the leakage of CSF [10]. Meanwhile, it is reported that the incidence is up to 75%.

L4-SP has high clinical value that can be demonstrated by all of those examples. However, the position of L4-SP is usually determined by the position of the highest point on both sides of PSIS. But the accuracy rate of palpation of L4-SP is 36%, which is too low to locate the body surface position of L4-SP accurately enough [11–13]. Besides, only a few reports have assessed the inter-individual variation of L4-SP. In order to understand the anatomy classification of L4-SP fully to improve the clinical effect, this research did numerical measure and statistic analysis of L4 on 500 subjects from the Chinese to investigate the relationship between L4-SP and body position line in tummy position by using 3D reconstruction. In that case, it could be helpful for improving the accuracy rate of L4-SP detection could be improved.

## Methods

### Patients

It should be excluded that 47 individuals with lumbar pelvic fracture, dysplasia, lumbar scoliosis, or other spinal pathologic abnormalities. Five hundred subjects, aging 18 to 75, participated in this study from 2014 to 2018. Due to the limited availability of data, sex, height, and weight were not included.

### Methods

In CT scans, the location of 500 subjects is standardized to guarantee the relationship between body position line and L4-SP. In the anteroposterior and lateral lumbar images, carry on 3D reconstruction to remove L4-SP and adjust the appropriate proportion for choosing the correct position, to ensure accurate measurement results. The spiral computed tomography (CT) scanner (Somatom Emotion; Siemens AG, Munich, Germany) was used with the following scan conditions: voltage, 130 kV; current, 180 mA; thickness, 0.75 mm; and matrix size, 512 × 512. The 3D images were stored on the picture archiving communication system (PACS version 4.0; DJ Health Union Systems Corporation, Shanghai, China).

**Fig. 1:**
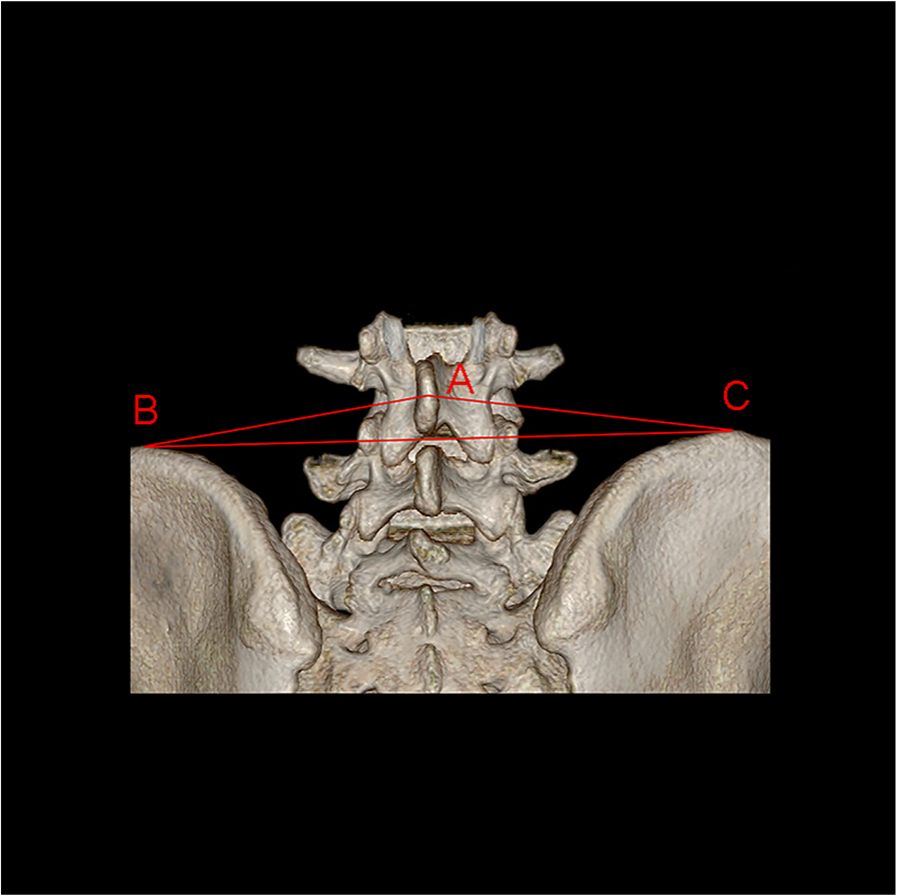
Measurements between L4-SP and posterior superior iliac spine (PSIS). Point A, the L4 spinous process; point B, the highest points of right side of PSIS; point C, the highest points of left side of PSIS

**Fig. 2:**
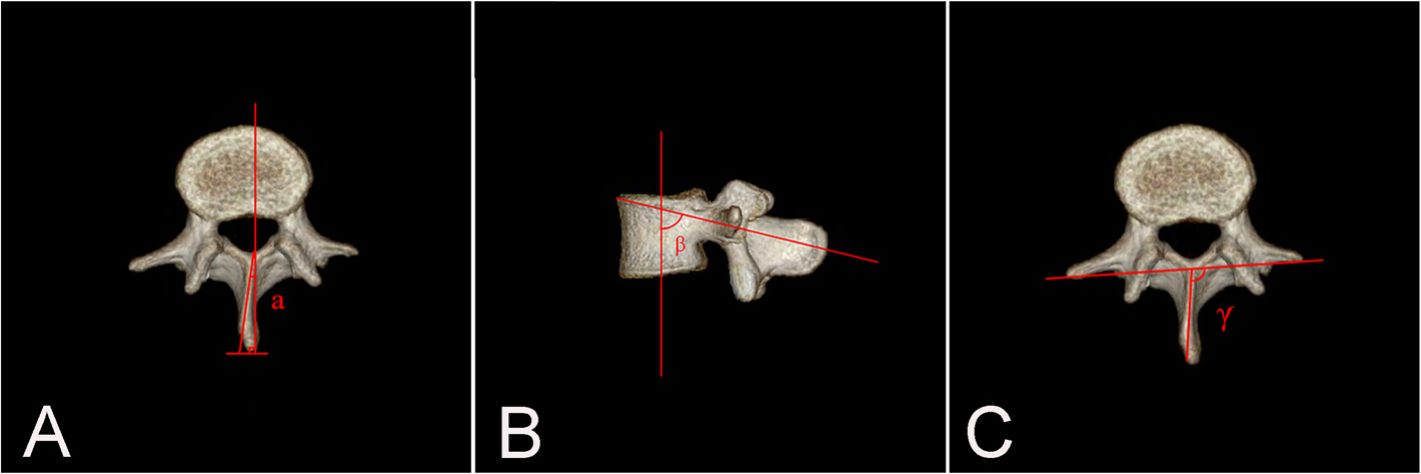
Measurements of L4. **a** ∠α, the angle between the axis of L4-SP and the median line of the vertebra in the superior aspect. **b** ∠β, the angle between the axis of L4-SP and the median line of the vertebra in the lateral aspect. **c** ∠γ, the angle between the SP axis and the line attached the transverse processes

The parameters were measured 3 times and recorded carefully by a doctor with 5 years of experience in 3D printing work for avoiding intra-observer and inter-observer variation. AB, the distance between point A and point B (point A the L4 spinous process, point B the highest points of the right side of PSIS); AC, the distance between point A and point C (point C the highest points of the left side of PSIS); and BC, the distance between point B and point C (Fig. 1). Angle-combined with AB and BC; what’s more, in order to find out the impact of the own factors of L4-SP on the results, measured the angle between the axis of L4-SP and the median line of the vertebra in the superior aspect (∠*α*) or in the lateral aspect (∠*γ*); and between the SP axis and the line attached the transverse processes (∠*β*) (Fig. 2). Length—the length of SP. The accuracy rate of palpation of L4-SP is 36% [11], and we adopted the same calculation: the number of spinous processes on AB divided by sample size multiply by 100%.

### Statistical analyses

A statistical analysis was proceeding with SPSS, version 20.0 (IBM Corp., Armonk, NY, USA). According to the formula *N* = *Z* × *P* × (1−P)/E, the sample size can be figured out (where *N* is a statistic, *Z* = 1.96; *P* is 0.5 as a probability value, *E* is 5% as an error value, confidence level is 95%). Through the above calculation, *N* = 500 > 384, and 500 scapulae could be adopted in the study. When it accorded with the normal distribution, the chi^2^ test and one-way ANOVA were adopted, and all data were presented with the mean and standard error. In addition, there is a *P* value < 0.05 as statistically significant.

### Ethics

All procedures were approved by the Ethical Committee of Affiliated Traditional Chinese Medicine Hospital of Southwest Medical University (SWMCTCM2017-0801).

## Results

Among 500 participants, three types were defined according to the distance of BC on the spine situating on different positions: 266 on L4-SP (type I, 53.20%), 16 above L4-SP (type II, 3.20%), and 218 below L4-SP (type III, 43.60%). Type I is the most common one among the Chinese population. All data were normally distributed, and measured relative data of the L4 SP based on classification were recorded in Table 1.

**Table 1:** The parameters of the L4-SP

Distribution	Type I	Type II	Type III
Numbers	266	16	218
Ratios (%)	53.20	3.20	43.60
BC (mm)	15.92 ± 1.30^b^	16.16 ± 1.18	15.56 ± 1.32
AB (mm)	8.02 ± 0.73	7.98 ± 0.76	7.89 ± 0.79
AC (mm)	7.96 ± 0.79	8.26 ± 0.81	7.97 ± 0.84
Angle (°)	173.00 ± 4.83^ab^	164.69 ± 5.50^b^	159.45 ± 8.39
α (°)	1.07 ± 3.05	1.00 ± 3.97	1.18 ± 3.33
β (°)	89.78 ± 5.28	90.00 ± 6.15	89.58 ± 3.44
γ (°)	74.46 ± 3.26	74.38 ± 3.65	74.16 ± 5.92
Length (mm)	3.06 ± 0.41	2.84 ± 0.52	3.07 ± 0.51

^a^*P* < 0.05 VS above the L4-SP

^b^*P* < 0.05 VS below the L4-SP

Significant differences were also observed in BC and the angle combined with AB and BC among different types. BC in type I (15.92 ± 1.30 mm) is longer than type III (15.56 ± 1.32 mm), while the angle combined with AB and BC in type I (173.00 ± 4.83°) is larger than that in type II (164.69 ± 5.50°) and type III (159.45 ± 8.39°). Comparing the length of the L4-SP to the left (AC) with the right (AB), there were no different significant among three types, and the same consequences also happened to three angles: *α*, *β*, *γ*. The number of spinous processes on AB was 266, according to the calculation, the accurate palpation rate for L4-SP is 53.20%.

## Discussion

Lumbar disk herniation, lumbar puncture, and spinal anesthesia were usually common situations, which involved the surface localization of L4-SP. The accuracy in identifying L4 using manual palpation by clinicians is limited due to the inter-individual variation in the morphology of L4-SP [2]. Most studies focus on the mechanisms of diseases affecting it. Even though, there were only a few reports about the inter-individual variation of L4-SP. The objective of the present study was to explore the anatomical features of L4-SP, which may facilitate to find the exact insertion point.

All data about 500 participants were collected by 3D of CT construction from 2014 to 2018. All patients were Han Chinese and aged 18 to 75. Thus, the information in this study had practical significance for the operation in China. By this way, Table 1 indicated that there were statistical differences in BC and the angles between three types. Among them 266 on L4-SP (type I, 53.20%), 16 above L4-SP (type II, 3.20%), and 218 below L4-SP (type III, 43.60%). This can be used to be one of the basis for determining the position of L4-SP. Unlike conventional X-ray analysis, CT provides detailed images of numerous types of tissue as well as the bones and blood vessels, which is a rapid and accurate procedure. In that case, based on anatomical parameters form CT to obtain a 3D model, they could locate it accurately and reduce the risk of operation [3, 5, 9]. When it came to some possible reasons, such as anatomic factors, the size of the lumbar spine changes with the development and the growth of human skeletons under the influence of genetic factors, and this kind of physiological diversity may affect the accuracy of palpation [4, 13–15]. Besides, degenerative changes occurring in the lumbar spine can be worse by the influence of patients’ lifestyles. The most common and serious transform was lumbar disk herniation, in broken disk herniation and retrograde lumbar disk herniation, which would cause broken fibrous ring, cartilage endplate, and nucleus burst out, and then resulting in the height of intervertebral disk changes. In addition, lumbar spondylolisthesis also had high incidence and influence the palpation accuracy, especially in different positions or sports. It is important to find the highest points PSIS to determine the accurate position of L4-SP [16]. On the account of normal physiological factors, the traditional palpation operation is used for normal state. However, there are 8 participants in the study that have 6 lumbar vertebrae, which cannot be adopted because of abnormalities.

As to the clinic factor, the degree of cooperation between patients and doctors can be an influential factor. Different levels of mistakes when different positions of the examinees took on, even experienced physician may make. If the 3D of CT construction was used to verify the position of the vertebral levels in patients, the risk of the devastating and permanent complications resulting from spinal cord injection can be reduced. In that case, it was clear that adequate knowledge of L4 morphology is necessary for the spinal surgeon in order to avoid damage to the vertebral arteries, spinal cord, or nerve roots during fixation interventions involving the posterior cervical spine. Hence, it can be more safer for patients with abnormal spinal anatomy and the morbidly obese, particularly in those who have difficulties in palpating anatomical landmarks [17, 18]. Therefore, there were a series of errors which may happen in clinical practice. The traditional palpation of L4-SP is not completely exact. The accuracy rate is only 53.20%, and the errors may cause serious consequences [19].

Nevertheless, there are still some limitations. Though the accuracy of the fourth lumbar spinous process palpation can be improved by 3D of CT reconstruction, the operative level of clinician is decisive. Above that, the samples in this research are limited to the southwest, and the sample size is not so adequate now. The image resources are limited when it comes to costs and radiation because it is far more practical that patients’ willingness to use radiographs, computed tomography, and MRI are reduced [18, 20].

## Conclusion

Among 500 participants, three types were defined according to the distance of BC on the spine situating on different positions. Among three types, type I is the most common one among the Chinese population. As an important position of the body, the accuracy rate of palpation of L4-SP has high clinical value. However, it is too low to locate the body surface position of L4 accurately enough now. This study demonstrated that the anatomy of L4 can be understood fully through 3D reconstruction technology and improve the clinical effect, so that the accuracy of SP’ detection can be authoritative.

## Data Availability

The datasets used and analyzed during the current study are available from the corresponding author on reasonable request.

## Abbreviations

L4-SP: Lumbar fourth spinous process
CT: Computed tomography
3D: Three-dimensional
PSIS: Posterior superior iliac spine
SA: Spinal anesthesia
